# Has Bipolar Disorder become a predominantly female gender related condition? Analysis of recently published large sample studies

**DOI:** 10.1186/s40345-020-00207-z

**Published:** 2021-01-04

**Authors:** Bernardo Dell’Osso, Rita Cafaro, Terence A. Ketter

**Affiliations:** 1 Department of Biomedical and Clinical Sciences Luigi Sacco, Department of Mental Health, University of Milan, ASST Fatebenefratelli-Sacco, Milano, Italy; 2 CRC Aldo Ravelli, Milano, Italy; 3 Department of Psychiatry and Behavioural Sciences, Stanford University, Stanford, CA USA

**Keywords:** Bipolar disorders, Gender differences, Prevalence, Female gender

## Abstract

Bipolar Disorders (BD) are disabling and severe psychiatric disorders, commonly perceived as equally affecting both men and women. The prevalence of BD in the general population has been growing over the last decade, however, few epidemiological studies are available regarding BD gender distribution, leaving unanswered the question whether the often reported increment of BD diagnosis could be gender specific. In fact, BD in female patients can often be misdiagnosed as MDD, leaving such women non correctly treated for longer times than their male counterparts. From this perspective, we searched literature for large sample (> 1000 subjects) studies published in the last decade (2010 onward) on BD patients. We included ten large sample studies that reported the gender distribution of their samples, and we therefore analysed them. Our results show a higher preponderance of female patients in every sample and sub-sample of BDI and BDII, supporting our hypothesis of an increase in BD diagnosis in females. BD in women presents with higher rates of rapid cycling, depressive polarity and suicide attempts, characteristics of non inferior severity compared to males; prompt recognition and adequate treatment of BD is therefore crucial to reduce risks and improve quality of life of affected women. In this regard, our results could lead the way for national or international epidemiological studies with the aim of more accurately assessing gender-specific prevalence of BD.

## Introduction

Bipolar Disorder (BD) has been traditionally included among psychiatric conditions with no gender difference in terms of lifetime prevalence in the general population, (Weissman et al. 1988; Mitchell et al. 2004; Grant et al. 2005; Merikangas et al. 2007) in contrast to unipolar Major Depressive Disorder (MDD), which has consistently shown a higher prevalence in the female gender. (Bijil et al. 2002; Kessler 2003; Leach et al. 2008) However, some studies showed specific differences in the clinical course as well as across various behavioural and social outcomes of BD in individuals with male versus female gender. For instance, early (pre-teen) age of onset of first mania, (Grant et al. 2005; Kennedy et al. 2005) substance use disorders, (Kessing 2004; Nivoli et al. 2011) and legal problems, (Baldassano et al. 2005) were reported to be more common in men, while rapid cycling, (Slyepchenko et al. 2017; Tondo and Baldessarini 1998) depressive episodes, (Diflorio and Jones 2010) mixed mania, (Diflorio and Jones 2010; Arnold et al. 2000) and attempted or completed suicides, (Nivoli et al. 2011; Clements et al. 2013) have been reported to be more frequent in women. Indeed, hypomania, and consequently BD type II (BDII), has commonly been more frequently associated with female gender, (Hendrick et al. 2000; Suppes et al. 2005; Altshuler et al. 2010) while no significant gender difference has been reported in lifetime prevalence of BD type I (BDI) (Diflorio and Jones 2010).

The overall limited epidemiological studies about BD conducted in the last decade showed increasing lifetime prevalence of BD in the general population, (Clemente et al. 2015) possibly due to better diagnostic instruments, especially with the implementation of DSM-IV diagnostic criteria (American Psychiatric Publishing 1994).

On the other hand, these studies did not evaluate the gender composition of recruited samples, leaving unanswered the question whether the better understanding of the disease lead to an improvement in its recognition in the female gender, in which misdiagnosis with unipolar MDD occurs frequently (Culpepper 2014; Lish et al. 1994; Hirschfeld et al. 2003). In this regard, the recognition of an epidemiological trend of gender specificity in BD could help physicians to pursue prompter diagnosis and implement more effective treatments, which are extremely important considering that use of antidepressants (i.e., misdiagnosis unipolar MDD) might lead to inefficacy for misdiagnosed (actually bipolar) depression, treatment-emergent (hypo)manic switching, onset of rapid cycling and, thus, negatively influence the course of the disease (Wehr and Goodwin 1987).

In light of these considerations, we aimed to analyse the gender composition of large samples studies on BD patients published in the last decade (from 2010 onward), to evaluate a possible modification in the trend of representation of the disease in the two genders.

## Methods

An electronic systematic review within the online databases PubMed, Medline, PsychINFO, and Cochrane Library was conducted up to May 2020. Keywords used were: “bipolar disorder” OR “mania” AND “gender” OR “gender difference(s)” AND “national cohort” OR “multicentric studies”. The results were filtered by year of publication (2011–2020) and number of patients included in the study (> 1000). We then proceeded to exclude studies not adherent to criteria and incomplete studies not providing information on sample composition in terms of (sub)diagnosis and gender distribution.

## Results

Ten clinical studies published in the last ten years, analysing data from more than 1000 bipolar patients per sample, met our inclusion criteria and are therefore presented and discussed here (Table 1). In aggregate, 47,878 patients with BD were included in these studies.

**Table 1.** Main large samples (> 1000 patients) studies on Bipolar Disorder published in the last ten years

Authors	Site	Sample	N° of BD patients recruited	Female patients (%)
Bareis et al. 2018	U.S.A.	STEP-BD (1999-2005)	3563	57,65%
Bobo et al. 2018	U.S.A.	Mayo Clinic Biobank (started in 2009)	1465 BD-I = 69,42% BD-II = 30,58%	60,75% BD-I = 58,60% BD-II = 66,10%
Buoli et al. 2019	Italy	RENDiBi Study (April 2014-March 2015)	1675 BD-I = 62,21% BD-II = 37,79%	57,40%
Crump et al. 2013	Sweden	National Registry (2001-2002)	6618	59,20%
Hayes et al. 2017	U.K.	Primary care electronics health records (THIN) (2000-2014)	17341	58,83%
Hou et al. 2016	Australia, Canada, Czech Republic, France, Germany, Italy, Japan, Poland, Romania,Spain, Sweden, Switzerland, Taiwan, U.S.A.	ConLiGen	2563	57,63%
Kalman et al. 2019	Austria, Australia, Canada, Czech Republic, France, Germany, Italy, Poland, Romania, Spain, Sweden, U.S.A.	ConLiGen, Bonn-Mannheim and PsyCourse	1995 (BD-I patients only)	55,10%
Karanti et al. 2015	Sweden	Swedish National Quality Assurance Register for Bipolar Disorder (BipoläR) (2004-2011)	7136 BD-I = 47,09% BD-II = 38,15% BD-NOS = 14,76%	61,27% BD-I = 57,30% BD-II = 64,95% BD-NOS = 64,42%
Vieta et al. 2013	Austria, Belgium, Brazil, France, Germany, Portugal, Romania, Turkey, Ukraine, Venezuela	WAVE-BD (April 2010- June 2011)	2896 BD-I = 68,70% BD-II = 31,3%	65,00%
Yoon et al. 2018	Korea	Korean HIRA-NPS (2013 sample only)	2626	58,80%

THIN = The Health Improvement Network; STEP-BD = Systematic Treatment Enhancement Program for Bipolar Disorder; RENDiBi = National Epidemiological Research on Bipolar Disorder; HIRA-NPS = Health Insurance Review and Assessment Service – National Patient Sample; WAVE-BD = Wide Ambispective of Bipolar Disorder; ConLiGen = The International Consortium on Lithium Genetics; BD-I = Bipolar Disorder type I

Two of these studies were conducted in Sweden, one including bipolar patients enrolled in the national registry of the years 2001–2002 (n = 6618), (Crump et al. 2013) while the other included bipolar patients from the Swedish National Quality Assurance Register for Bipolar Disorder (BipoläR), recruiting patients from 2004 to 2011 (n = 7136) Karanti et al. 2015). Another study was conducted in the United Kingdom (U.K.), analysing data from patients included in “The Health Improvement Network” (THIN), from 2000 to 2014 (n = 17,341); (Hayes et al. 2017) THIN is a database of primary care electronics health records representative of the general U.K. population in terms of age, gender, medical conditions, and death rates, and has been validated for research purposes against experimental and observational evidences. Similarly, one study was conducted in Korea and was based on the Health Insurance Review and Assessment Service – National Patient Sample (HIRA-NPS) records of 2010, 2011 and 2013; importantly, validity and representativeness of HIRA-NPS data have been extensively demonstrated (Kim et al. 2014). However, as there is no continuity between data for different years, in order to avoid duplicates, we included in our review only data from 2013 (n = 2626) (Yoon et al. 2018). Two studies were based in the United States of America (U.S.A.); one of them analysed data from the “Systematic Treatment Enhancement Program for Bipolar Disorder” (STEP-BD), a nationwide database collected from 1999 to 2005 (n = 3563) (Bareis et al. 2018). The other was based on the Mayo Clinic Biobank, started in 2009 and including patients recruited until 2018 (n = 1465) Bobo et al. 2018). Yet another study was an Italian study collecting data in the context of the National Epidemiological Research on Bipolar Disorder (RENDiBi) project, in which patients were recruited from April 2014 to March 2015 (n = 1675) (Buoli et al. 2019). Finally, three studies were based on multinational multicentric databases; one was the Wide AmbispectiVE study of the clinical management and burden of BD (WAVE-BD), recruiting patients from April 2010 to June 2011 (n = 2896) (Vieta et al. 2013). Another study collected data from the ConLiGen database (n = 2563) (Hou et al. 2016), while the last one included data collected from ConLiGen, Bonn-Mannheim and PsyCourse databases (n = 1995, BDI patients only) (Kalman et al. 2019).

**Fig. 1:**
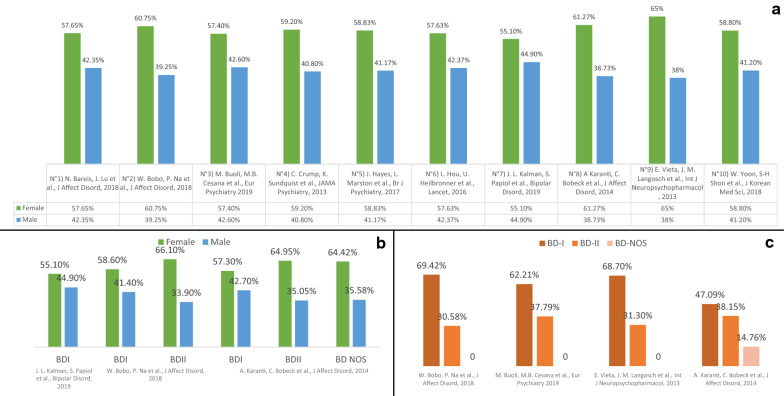
Gender distribution in Bipolar Disorder as shown in the main large sample studies of the last ten years. **a** Graphic representation of gender distribution in 9 main large sample studies, reported as percentage of female and male participants upon the total number of BD patients; **b** percentages of females and males in BD type-specific samples; **c** gender distribution of three samples, confronted with the BD type-specific composition of the samples. BDI = Bipolar Disorder type I; *BDII * Bipolar Disorder type II.

Nine out of ten studies analysed samples of both BDI and BDII patients, while one study only analysed a sample of BDI patients. All the evaluated studies described the general distribution of their samples in terms of gender, reporting a higher prevalence of female patients, with female patient proportions ranging from 57∙4% (RENDiBi Study) to 65∙0% (WAVE-BD) (Fig. 1a). Of these, three studies (Mayo Clinic Biobank, RENDiBi Study, WAVE-BD) described the distribution of their samples in terms of BDI and BDII (Mayo Clinic Biobank 69∙4% of BDI, RENDiBi Study 62∙2% of BDI, WAVE-BD 68∙7% of BDI), while one of them (BipoläR) further categorised patients in BDI (47∙1%), BDII (38∙1%), and Bipolar Disorder – Not Otherwise Specified (BD NOS) (14∙8%). In these four studies, a higher prevalence of BDI diagnosis among women can be found (Fig. 1c). Moreover, the Mayo Clinic Biobank study reported the gender distribution of the sub-samples BD-I (F = 58∙6%) and BD-II (F = 66∙1%), while the BipoläR study reported the gender distribution in the three sub-samples BDI (F = 57∙3%), BDII (F = 64∙9%), and BD NOS (F = 64∙4%). As shown, both these studies reported a higher prevalence of female versus male patients in every sub-sample (Fig. 1b).

Finally, one study evaluated a sample of BDI patients only, reporting a higher prevalence of female versus male patients (55∙1%).

## Discussion

As shown in the results, 100% (ten out of ten) of our selected large samples studies (n > 1000) published in the last ten years (2011–2020), showed a predominance of female gender in their samples of bipolar patients. These results could support our hypothesis of a relatively recent increase in BD prevalence among individuals with female gender. Even though data published in the Global Burden of Disease Study of 2013 seem to support the findings of a higher prevalence of BD in the female gender (Ferrari et al. 2016), our results seem to be in contradiction with previously published literature about BD prevalence in the two genders (Weissman et al. 1988; Mitchell et al. 2004; Grant et al. 2005; Merikangas et al. 2007; Diflorio and Jones 2010). Causes for this difference could lay in the size of the samples analysed, being that the majority of the studies presented in literature analyse much smaller samples. Moreover, one could argue that the higher percentage of females diagnosed with BD-II could interfere with the gender distribution of the samples analysed, and therefore be the cause of our findings. However, of note, Bobo et al. (Bobo et al. 2018) Buoli et al. (Buoli et al. 2019) Karanti et al. (Karanti et al. 2015) and Vieta et al. (Vieta et al. 2013) had a higher proportion of BDI patients in their samples, and Kalman et al. (Kalman et al. 2019) only had BDI patients in their sample. Findings of a higher prevalence of female patients in these four studies could further support our hypothesis, being BDI the type of BD traditionally more commonly associated with equal prevalence in the two genders (Mitchell et al. 2004; Grant et al. 2005).

Even though not all of the studies discussed here are epidemiological, all of them represent the gender distribution of wide national or international coverage studies, with high numbers of patients, and therefore report a fairly realistic picture of the distribution of the disease in the population to which they refer. Buoli et al. (Buoli et al. 2019) analysed the sample of a nationwide epidemiological study, Hayes et al. (Hayes et al. 2017) and Yoon et al. (Yoon et al. 2018) recruited patients from samples representative of the national population, while Crump et al. (Crump et al. 2013) recruited patients from the Swedish National Registry, which included every person at least 20 years of age that lived in Sweden for at least two years as of January 1, 2003.

Overall, the gender distribution of BD reported in these studies, along with the increase of BD diagnosis in the last decade, (Clemente et al. 2015) could be signals of more efficient BD diagnosis, especially in the female gender. Since female gender in BD has already been associated with higher rates of rapid cycling, (Carvalho et al. 2014) dysphoria, and lifetime prevalence of depressive episodes, (Morgan et al. 2005) and suicide attempts, (Nivoli et al. 2011) indicating that BD in women can exhibit characteristics of non-inferior severity of illness and reduced quality of life, compared to their male counterparts, prompt recognition of BD in female patients is crucial in order to provide more adequate treatment. In particular, the frequent observation of a prevalent depressive polarity in female patients (Nivoli et al. 2011), with more frequent first manifestation of BD through a major depressive episode (MDE) and the higher lifetime number of MDEs, represent significant challenges for clinicians who may misdiagnose them as affected by unipolar MDD and consequently treat them with antidepressants rather than with mood stabilizers and atypical antipsychotics, a treatment that, particularly if administered in monotherapy, could concur in the onset of rapid cycling (Valentí et al. 2015). In this regard, although some studies in literature support the use of antidepressants as adjunctive short-term treatment in bipolar depression (Gijsman et al. 2004), data on the topic are inconsistent (Sachs et al. 2007), and antidepressants alone yield a high risk for manic switch and therefore mood stabilizers are still the cornerstone of bipolar disorders treatment (Price and Tyrka 2007).

Moreover, BD in women poses a significant burden on pregnancy, with a higher risk of adverse pregnancy outcomes and a higher frequency of relapses during the postpartum period, (Bodén et al. 2012; Wesseloo et al. 2016; Viguera et al. 2011) making prophylactic medication during pregnancy fundamental in order to maintain mood stability postpartum (Wesseloo et al. 2016). Of note, the correlation between the physiology of pregnancy and BD relapses could underlay hormonal and endocrinological influence on BD symptoms and presentation, as it has already been demonstrated for unipolar MDD (Studd and Panay 2009; Joffe 2011; Graziottin and Serafini 2009). The extensively reported correlations between sex hormones and mood dysregulation in women, could be significant also for BD, not only in terms of prevalence, but also for providing better care for female BD patients, which could benefit from personalized and hormonal treatments, as it already happens for unipolar MDD (Graziottin and Serafini 2009). It has already been suggested, in this regard, that women treated for BD show greater degrees of menstrual-entrained mood fluctuations, which could be mitigated by mood stabilizing treatments (i.e. Lamotrigine) (Robakis et al. 2015).

In conclusion, as BDs represent highly comorbid, disabling, difficult-to-treat and life-threatening conditions, clinicians should be aware of the recently reported increase in the prevalence of such conditions in the female gender and pay additional attention in the differential diagnosis with unipolar MDD, particularly when the first manifestation of BD is represented by a MDE. Prompter diagnosis and appropriate treatment for females with BD, in fact, could reduce acute inefficacy, longer-term relapses, suicide attempts, treatment-emergent affective switching, and onset of rapid-cycling.

Although in the ten large samples studies analysed here it is possible to identify a higher prevalence of female BD patients, the majority of these studies are not epidemiological, and their samples are collected over many years, many in BD Specialty Clinics, which commonly report having more female than male patients. In this regard, female patients, especially in younger generations, are more often willing to seek help for their symptoms, leading to a possible sampling bias. On the other hand, the often reported comorbid substance use disorders in male patients might concur in the fewer men seeking treatment in Bipolar Disorder Specialty Clinics, enhancing this possible sampling bias. Adding to this, female patients often seek help for ruminative thinking associated to life events stressors (Michl et al. 2013), and ruminative thinking is often interpreted as a sign of depression (McLaughlin and Nolen-Hoeksema 2011). To avoid these confounding factors, nationwide or international epidemiological studies are needed in order to confirm our preliminary observation.

Being the samples analysed here only based on longitudinal diagnosis of BD, another limitation of our study is the lack of data on mood presentation at the time of the studies, therefore not addressing one of the greatest differences in BDs presentation between genders.

Moreover, the studies analysed here recruited samples from specific areas or nations. It could be, from this point of view, that our conclusions cannot be extended to the international population but are only true for the specific regions of interest. It is well known, in fact, that the gender distribution of the general population varies across the world, with some countries (especially eastern countries) with a predominant male population. It is therefore possible that, for these countries, further and more specific studies on the subject should be carried out.

Lastly, even though the higher number of reported BD diagnosis in the last years could be connected to better diagnostic criteria implemented in the most recent editions of the DSM, (American Psychiatric Publishing 1994) on the other hand, more comprising diagnostic criteria could misleadingly help diagnose BD when it is not present, and therefore lead to a selection bias. In this regard, a right diagnose and a thorough follow-up are extremely important in order to provide the right treatment to patients presenting with depressive symptoms.

## Data Availability

All data generated or analysed during this study are included in this published article.
